# Supplementary material for: Myosin VIII associates with microtubule ends and together with actin plays a role in guiding plant cell division
Source: eLife. 2014 Sep 23;3:e03498. doi: 10.7554/eLife.03498 (PMC4171706; doi:10.7554/eLife.03498)
Supplement: Figure 4—source data 1. — DOI: http://dx.doi.org/10.7554/eLife.03498.012 [file elife03498s001.docx]

CLUSTAL O (1.2.1) multiple sequence alignment

PpMyo8B -MFP-LKSSGPRSTLEEMLDLFRTDDKQEK-ESMDNGEEARPPPLPVRPASR--ARLPSS

PpMyo8C -MFP-LKSSGPRSTLEEMLDLFRTNDTQEEGTSKDNGEEARPPPLPARPTSR--ARLPSS

PpMyo8E -MFS-SVGNDSRTPVRGVQDFTTAEYIEEDDMLSDETENDVVLSAPSMPTSR--ARLSSS

PpMyo8A -MYS-TNGIEGRSTLEKMLDFMKTGDIEESEITSDETYAD-LPPLPLRPSSR--ARLPSS

PpMyo8D -MLS-SNACEGRSTLEEMLDLISTGDTEEREVSSDDTQED-LLPLPSRPTSR--ARLPSS

NbMyo8A -MLSVSAPSRTRSSLEEMLESLKQRDENEK-------PKDIPPALPTRPKSASRTRPPSP

NbMyo8G -MLSVSAPSRTRSSLEEMLESLKQRDEDEK-------PKDIPPALPTRPKSASRTRXPSP

NbMyo8C -MLSVSPNSMARSSLEEMLETLRRRDENEK-------PKDLPPALPARPKLKSKTRPPSP

NbMyo8E -MLSVSPNSMARSSLEEMLETLRRRDENEK-------PKDLPPALPARPKLKSKTRPPSP

NbMyo8B ----------------------------------------MAQ----R-VKG--APSLQS

NbMyo8D ----------------------------------------MAQ----R-VKG--APSLQS

AtMyo8B ----------------------------------------MA-------HKV--KASFQS

AtMyo8D ----------------------------------------MS-------QKV--TPFMQS

AtMyo8A ---------MMKSSVKEILESLRLLDSSER-------SSS----LPSPSTFR--APMPLI

AtMyo8C MMLSASPNTLAKSSLEEMLESLRQKDECDR-------PKDMPPALPSRPNSR--ARLPSA

PpMyo8B VRARKHQVVAMVKLISSSERSVDVVQGDVAVDAPAVNTVLCPVTDCLDVDDDQVSKPERC

PpMyo8C VRARKQQVIAAVKLAPASKIPVDVAKENLATDSLDADKALPFVIDCLNRDDDQVPKPESF

PpMyo8E MRAKKAPGACIDNLAIPSKGSGMLLKENIALGSPISNLITAVDPAGL--ASRCLSKP---

PpMyo8A MGAKKALGACLDSIVLSSNESEAFKE-NIAVGSPIVNLVAPADPAAL--ASKS-------

PpMyo8D VRAKKALGVCLDNIVPSSNGSAALSKEIVFLGSPIANLTVPSDPLSL--APKS-------

NbMyo8A KRTLPNNTT--GNRSVVE------LENG--------------------------------

NbMyo8G KRTLPNNTT--ENRGVVE------LENG--------------------------------

NbMyo8C KRTLPNILG--I-KGDVG------LEKN--------------------------------

NbMyo8E KRTLPNSFG--I-KGDVQ------LEKN--------------------------------

NbMyo8B IKSLPVGYA--FDLNKSE----AVNHR---------------------------------

NbMyo8D IKSLPVGYA--FDLNKSE----AVNHR---------------------------------

AtMyo8B LKTMPADYR--FLGSPIS----DHLETN--------------------------------

AtMyo8D LKSLPADYR--FDGSPVS----DRLENS--------------------------------

AtMyo8A RQSLPAKFR--NAIS---------LESK--------------------------------

AtMyo8C RRSLPANFN--VSSV---------MEDQ--------------------------------

: .

PpMyo8B QERPVNHPPAIVIPDDQALQNGHMAFETNVTATNCTKMSESQEITASLTNPAAISVGKQR

PpMyo8C RERISDHLSATKAHADQAPQNGHTVVESTLNTPNCTNKSDLEESKAPWTCPAVVSVGRPG

PpMyo8E --------------FDLASENSHAPLAEREIVANYGSLPNPQFVSSPTIMPTLFTPAKES

PpMyo8A ----------------VTCTNVHTPLAERVGTALNESFASPQLASSPSIIPDVFTPADQV

PpMyo8D ----------------FASENGYTPLAKGEDSANNESFASPNLAYSPTIIPDVLMQSDEV

NbMyo8A ------------------------------------------------------------

NbMyo8G ------------------------------------------------------------

NbMyo8C ------------------------------------------------------------

NbMyo8E ------------------------------------------------------------

NbMyo8B -------------------------MASNGAVSKNGELSS-------------------G

NbMyo8D -------------------------MASNAAVYKNGELSS-------------------E

AtMyo8B -----------------------------LITPPNGHLKN-------------------G

AtMyo8D --------------------SGASVRLTNSNVPRKGGLRN-------------------G

AtMyo8A --------------------TI--------------------------------------

AtMyo8C --------------------NGSVVSVTPAVEAESERKEE-------------------G

PpMyo8B VGEEEAELHSNKLLVDVRGTANGSVVDDGAAVEQSDFSALPPEQLA------------LL

PpMyo8C GSEEEAELDSRNLSFDFGGMPNGSLADEETELGRTDLSALVPEQPA------------L-

PpMyo8E -------NAQMGSIFDDR--INVSGDNRGFSREQSTFSFLTAQEPP-------APQTPGA

PpMyo8A -------RSSGTLSFDQR--LDACG------AQESSFSFLTAQESS-------TPETPLP

PpMyo8D -------RRSRTLSFGER--LNACS------TQERSFSFLTAQESS-------TPHTPLP

NbMyo8A --KKEEVKGKRGNMFGAKKGKEMIM-----EFSESPYVNSFSVEKEYRQRFWEKDGAKL-

NbMyo8G --KKEEVKGKRGNMFGAKKGKEMVV-----EFSESPYVNSFSVEKEYRQRFWEKNGAKL-

NbMyo8C --SSKILEES--------KGFQRNA-----NFGA--------------------------

NbMyo8E --SSKKLEEF--------KGLQMNA-----SFGG--------------------------

NbMyo8B VNG----------------SADG-------YIDESPYGRLNFS----------VDERPS-

NbMyo8D ANG----------------NADG-------YIDESPYGRLNFS----------VEESPS-

AtMyo8B VNGTASSVG----------GMDS-------VNEDSPYSVRSIL----------NGERS--

AtMyo8D VSRTDTAAG---------------------DSEDSPYSGHGVF----------VEEQSL-

AtMyo8A -EKEDKDWSTEQ-------I---------------TQSAEKEK----------TGN--E-

AtMyo8C VKRKEKDLGVKRNSFGSKKMRTG-------LRSESPYAAEKEE----------EGVKIS-

PpMyo8B QIPSLQSPARTPTSPAPSRKWIDDGVLRLRKNLRVWCLTS-ENIWICGTIISVE--DAEA

PpMyo8C -------PPHSPSSPAPSRKWKDDGVLRLKKNLRIWCLTS-DCMWIPGVIISVE--DTEA

PpMyo8E ENF----ALHPATTPSSGKKWRDDGTLRLKKNLRVWFLSS-DYNWIAGTVITIE--DTEA

PpMyo8A QTPVLENTALPVTTPSSGKKWKDDGTLRLKKNLRVWCLTS-EYNWIAGTVVSAEDKDTEA

PpMyo8D QNPLVEDTSLPVTTPSAGKKWKDDGILRLKKYMRVWCLSS-EYNWIAGTIVSAENKDAEA

NbMyo8A LD--NNNRVHYSLPKFRENEWNDNISYFIEKKLRVWCHLK-NRQWEAGQIQSTFGDTAS-

NbMyo8G LD--NNNRVHCSLPKFREDEWNDNISYFIEKKLRVWCHLK-NRQWEAGQIQSTSGDKAS-

NbMyo8C --------------YKKVKGMEPNINYFINKKLRIWCRLR-NGQWVSGQVQSSSGDKAT-

NbMyo8E --------------YKKVKGMEPNINYFINKKLRIWCCLR-NGQWVSGQVQSSSGDKAT-

NbMyo8B -----SCDDDLRTNAFASSKWSDTTSYMTKKKLHSWFQLP-DGNWELATIISKSGNEVL-

NbMyo8D -----SCDDDLRTNAFASSKWSDTTSYLTKKKLHSWFQLP-DGNWELATIISKSGNEVL-

AtMyo8B --SIGDGDSILPLPESNDRKWSDTNVYARKKVLQFWVQLP-NGNWELGKIMSTSGEESV-

AtMyo8D TDDVDSGAATMPLPQSDERRWSDTSAYARKKILQSWIQLP-NGNWELGKILSTSGEESV-

AtMyo8A VVKI-STAQMSRAKNSHDPEWINSAEYFVREKLCVWCRVAANGQWHLGKIHSTSSSDDVC

AtMyo8C IAKV-SLVENTEEHNKPESEWNNNVEYFIKKKLRVWCRVS-NGQWQLGKIQSTSADTSL-

.: : * : * . : :

PpMyo8B VVWTSDREEIQVSVTKLLPANPAFLEGVDDLIKLSYLNEPSVLHDLDYRYSKDQIYTKAG

PpMyo8C VVRTSDRQEIRVSATKLLPANPAFLEGVDDLIKLSYLNEPSVLHDLDYRYSKDQIYTKAG

PpMyo8E VVRTPDQLMIKVNASSLQPANPEILEGVFDLIKLSYLNEPSVLHNLAFRYAKDKIYTRAG

PpMyo8A MVRTADHKVIRVNVTRLQPANPDILEGVYDLIKLSYLNEPSVLHNLDFRYEQDKIYTKAG

PpMyo8D MVRTADHQIIRVNVTRLKPANPDILEGVHDLIKLSYLNEPSVLHNLEFRYAHDKIYTRAG

NbMyo8A -VLLFDGSVVAVPIGELLPANPDILQGVDNLIQLCYLNEPSVVHNLEHRYHQDRIYTKAG

NbMyo8G -VLLSDGSVVAVPIGELLPANPDILQGVDNLIQLCYLNEPSVVHNLQHRYHQDRIYTKAG

NbMyo8C -VLLSDRSFVTVPVGELLPANPDVLEGVDDLMQLSYLNEPSVLHNLQHRYARDIIYSKAG

NbMyo8E -VLLSDRSVVTVPVGELLPANPSVLEGVDDLMQLRYLNEPSVLHNLQHRYARDIIYSKAG

NbMyo8B -ISLSEGKVLKVKADDLLPANPDILDGVDDLMQLSYLNEPSVLYNLQYRYNRDMIYTKAG

NbMyo8D -ISLSEGKVLKVKADDLLPANPDILDGVDDLMQLSYLNEPSVLYNLQYRYNRDMIYTKAG

AtMyo8B -IVVTEGKVLKVKSETLVPANPDILDGVDDLMQLSYLNEPAVLYNLEYRYNQDMIYTKAG

AtMyo8D -ISLPEGKVIKVISETLVPANPDILDGVDDLMQLSYLNEPSVLYNLNYRYNQDMIYTKAG

AtMyo8A VMLSANDDVVKVAMEEIFPANPEILEGVEDLTQLSYLNEPSLLYNLRVRYSQDLIYSKAG

AtMyo8C -VMLSTANVVKVSTEELFPANPDILEGVEDLIQLSYLNEPSVLYNLRVRYLQDVIYSKAG

: : * : **** .*:** :* :* *****:::::* ** :* **::**

PpMyo8B PVLIAVNPFKKIHIYGEDIMQAYRDRTSASSQPHVYMIAGSAFGAMMKEGINQSIIISGE

PpMyo8C PVLIAVNPFKKIPIYGEDIVQAYQKAAPASSQPHVYMVADSAFGAMMKEGINQSIIISGE

PpMyo8E PVLIAVNPFKKVPIYGPDSVQAYQKRTPESSHPHVYMTADTAFNAMMRDGINQSIIISGE

PpMyo8A PVLIAVNPFKEISIYGPNNILAYRNRTSESTYPHVYMTADTAFKAMIRDGINQSVIISGE

PpMyo8D PVLIAVNPFKQIPIYGPDNVQAYQRRTSESSHPHVYMTADSAFKAMVRGGINQSIIISGE

NbMyo8A PVLIAVNPFKEIQLYGNEHITAYRQKLLD--DPHIYSVADTAYSQMMEDEINQSIIISGE

NbMyo8G PVLIAVNPFKEIQLYGNEHITAYRQKLLD--DPHIYSVADAAYSQMMEDEINQSIIISGE

NbMyo8C PVLIAINPFKDIQLYGDEFVTAYRQKLLN--DPHVYFIADTAYDRMMEDEISQSIIISGE

NbMyo8E PVLIAINPFKDIQLYGDEFVTAYRQKLLN--DLHVYSIADTAYDRIMEDEISQSIIISGE

NbMyo8B PVLVAVNPFKKVSLYGNEYIEAYKRKSIE--SPHVYAITDMAIREMVRDEVNQSIIISGE

NbMyo8D PVLVAVNPFKKVSLYGNEYIEAYKRKSIE--SPHVYAITDMAIREMVRDEVNQSIIISGE

AtMyo8B PVLVAVNPFKEVPLYGNRNIEAYRKRSNE--SPHVYAIADTAIREMIRDEVNQSIIISGE

AtMyo8D PVLVAVNPFKEVPLYGNRYIEAYRKKSNE--SPHVYAIADTAIREMIRDEVNQSIIISGE

AtMyo8A PVLIAVNPFKNVQIYGEEFLSAYQKNALD--APHVYAVADAAYDDMMREEKNQSIIISGE

AtMyo8C PVLIAVNPFKNVEIYGNDVISAYQKKVMD--APHVYAVADAAYDEMMREEKNQSLIISGE

***:*:****.: :** : **: *:* : * ::. .**:*****

PpMyo8B SGAGKTETAKIAMQYLAALGGGS-GIEDEILQTNPILEAFGNAKTSKNDNSSRFGKLIDI

PpMyo8C SGAGKTETAKIAMQYLAALGGGS-GIEDEILQTNPILEAFGNAKTSRNDNSSRFGKLIDI

PpMyo8E SGAGKTETAKIAMQYLAALGGGG-GLEDEILQTNPILEAFGNAKTLRNDNSSRFGKLIDI

PpMyo8A SGAGKTETAKITMQYLAALGGGG-GLEDEILQTNPILEAFGNAKTLRNDNSSRFGKLIDI

PpMyo8D SGAGKTETAKIAMQYLAALGGGG-GLEDEILQTNPILEAFGNAKTLRNDNSSRFGKLIDI

NbMyo8A SGSGKTETAKYAIEYLAMISGGNNRIESEVLQTSCILEAFGNAKTPRNNNSTRFGKLIEI

NbMyo8G SGSGKTETAKYAIEYLAMISGGNNRIESEVLQTSCILEAFGNAKTPRNNNSTRFGKLIEI

NbMyo8C SGSGKTETAKIAMEYLAMIGGGRNAIEREVLQTSYILEAFGNAKTSKNNNSSRFGKLIEI

NbMyo8E SGSGKTETAKIAMEYLAMIGGGRNAIEREVLQTSYILEAFGNAKTSKNNNSSRFGKLIEI

NbMyo8B SGAGKTETAKIAMQYLAALGGGS-GIEDEILKTNPILEAFGNAKTLRNDNSSRFGKLIEI

NbMyo8D SGAGKTETAKIAMQYLAALGGGS-GIEDEILKTNPILEAFGNAKTLRNDNSSRFGKLIEI

AtMyo8B SGAGKTETAKIAMQYLAALGGGS-GIEYEILKTNPILEAFGNAKTLRNDNSSRFGKLIEI

AtMyo8D SGAGKTETAKIAMQYLAALGGGS-GIEYEILKTNPILEAFGNAKTLRNDNSSRFGKLIEI

AtMyo8A SGAGKTETAKYAMQYLEALGGGSFGVENEILKTNCILEAFGNAKTSRNDNSSRFGKLMEI

AtMyo8C SGAGKTETAKFAMQYLAALGGGSCGVEYEILKTTCILEAFGNAKTSRNANSSRFGKLIEI

**:******* :::** :.** :* *:*:*. ********** :* **:*****::*

PpMyo8B HFDESGKICGAIIETYLLEKSRVVQQAEGERSYHVFYQLCAGADESLRDLLRLRSAKEYR

PpMyo8C HFGESGKICGANIQTYLLEKSRVVQQAEGERSYHVFYQLCAGADESLRVRLSLRPAKEYR

PpMyo8E HFDRSGRICGAYIHTYLLEKSRVVKQAEGERSYHVFYQLCAGANRPLQERLHLKSAKEYR

PpMyo8A HFDRAGKICGAKIQTYLLEKSRVVQQAEGERSYHIFYQLCAGADTALRERLHLKSAKEYK

PpMyo8D HFDRTGKICGAKIQTYLLEKSRVVQQAVGERSYHVFYQLCAGADTALRERLYVRSAKEYR

NbMyo8A CFSAEGGICGANVQTFLLEKSRVVQLARGERSYHIFYQLCAGAPSALRDKLKLKGASDYN

NbMyo8G CFSAEGGICGANIQTFLLEKSRVVQLARGERSYHIFYQLCAGAPSALRDKLKLKGASDYN

NbMyo8C HFSATGRICSAKIQTLLLEKSRVVQLGNGERSYHIFYQLCAGAPPTLRDKLKLKGASEYK

NbMyo8E HFSATGRICSAKLQTLLLEKSIVVQLVNGDRSYHIFYQLCAGAPPTLRDKLKLKGASEYK

NbMyo8B HFSETGKISGANIQTFLLEKSRVVQCSEGERSYHIFYQLCAGAPGALKEKLNLKDVSEYN

NbMyo8D HFSETGKISGANIQTFLLEKSRVVQCSEGERSYHIFYQLCAGAPGALKEKLYLKDVSEYN

AtMyo8B HFSETGKISGAQIQTFLLEKSRVVQCTEGERSYHIFYQLCAGASPTLREKLNLTSAKQYN

AtMyo8D HFSESGKISGAQIQTFLLEKSRVVQCAEGERSYHIFYQLCAGASPALREKLNLTSAHEYK

AtMyo8A HFSAKGKICGAKLETFLLEKSRVAQLCNGERCYHIFYQLCAGASPILKERLKIKAASEYN

AtMyo8C HFSAMGKICGAKLETFLLEKSRVVQLFNGERSYHIFYELCAGASPILKERLKLKTASEYT

* * *..* :.* ***** *.: *:*.**:**:***** *: * : . :*

PpMyo8B YLSQSSCMSIDNVDDAEQFQRLRKAMNVVQICKEDQQKVFELLSAVLWLGNIVFRVSEPD

PpMyo8C YLNQSSCLSIDNVDDAKQFRHLRNAMSVVQICEEEQEQVFELLSAVLWLGNITFCVVEPD

PpMyo8E YLSQSNCLSIDNVDDAEKFQNLRSAMNVVDISKEDQEQSFEMLSAVLWLGNITFSVVEYD

PpMyo8A YLNQSRCLYIDNVDDAKNFQHMKSAMDVVQISVEDQEQAFKMLAAVLWIGNITFHVVEND

PpMyo8D YLDQSSCLSIEKVDDAKNFQHLKSALNVVQISQEDQEQIFEMLSAVLWIGNITFRVIDHD

NbMyo8A FLNQSDCLVIHDVDDAKKFHILVKALNTMGMSERDQEHAFQMVAVVLWLGNITFQAIGSE

NbMyo8G FLNQSDCLVIHDVDDVKKFHMLVKALNTMGMSERDQEHAFQMVAVVLWLGNITFQATGSV

NbMyo8C YLNRSDCLVIHDIDDAEEFRKLMEALNTFRIAERDKEHVFQMVASVLWLGNITFEVIDNA

NbMyo8E YLNRSDCLVIHDTDDAEEFRKLMEALNAFRISERDQEHVFQMSASVLWLGNIKFEVIDNA

NbMyo8B YLRQSNCYSISGVDDAEQFRIVMEALNVVHISKEDQESVFSMLAAVLWLGNISFTSVDNE

NbMyo8D YLRQSNCYSISGVDDAEQFRIVMEALNVVHISKEDQESVFSMLAAVLWLGNISFTSVDNE

AtMyo8B YLKQSNCYSINGVDDAERFHAVKEALDIVHVSKEDQENVFAMLAAVLWLGNVSFTIIDNE

AtMyo8D YLGQSNCYSINGVDDAERFHTVKEALDIVHVSKEDQESVFAMLAAVLWLGNVSFTVIDNE

AtMyo8A YLNQSNCLTIDRTDDAQKFHKLMEAFNIVQIPQEYQERTFALLAAVLWLGNVSFEVIDNE

AtMyo8C YLSQSDCLTIAGVDDAQKFHKLLEAFDIVQIPKEHQERAFALLAAVLWLGNVSFRVTDNE

:* :* * * **.:.*: : .*:. . : . :: * : : ***:**: *

PpMyo8B NHVVVVDNEAVEIAAALLGCEVDKLVTALYSRRIRAGGDTIVQRLTLSQATDSRDALAKA

PpMyo8C NHVVVKDKEAVEMAATLLHCDAGKLVIALTTRRIRAGGDIIVQRLTLSQATDSRDALAKA

PpMyo8E NHVVVDENEAVKVAAALLHCECSDLIAALSTRRIRAGGDHIIQRLTLTQATDSRDALAKA

PpMyo8A SYVVVDESEAVNVAAGLLHCKSNALVAALSTRRIRVGGEEIVQRLTFAQANDSRDALAKA

PpMyo8D NHVVVNENEAVNVAAGLLHCKSSALVAALSSRRIRVGGEEIVQRLTLTQANDSRDALAKA

NbMyo8A NNVEVAQSEAVINASSLLGCSANDLMLALSTRRMQTGKDKVVKSLTMQQAIDTRDALAKF

NbMyo8G NNVEVAQSEAVINASSLLGCSANDLMLALSTRRMQTGKDKVVKSLTMQQAIDTRDALAKF

NbMyo8C SHVEVVQSEAVTNAASLIGCRVNDLMLALSTRQIQVGKDKVAKSLTMEQATDRRDTLAKF

NbMyo8E SHVEVAQSEAVTNAASLIGCRVNDLMLALSTRQIQVGKDKVAKSLTMEQATDRRDTLAKF

NbMyo8B NHAEPVVDEGLTTVSTLIGCGLEELKLALSTRKMRVRNDDIVQKLTLSQATDTRDALAKS

NbMyo8D NHAEPVVDEGLTTVSTLIGCGVEELKLALSTRKMRVRNDDIVQKLTLSQATDTRDALAKS

AtMyo8B NHVEPEPDESLSTVAKLIGCNINELKLALSKRNMRVNNDTIVQKLTLSQAIDARDALAKS

AtMyo8D NHVEPVADESLSTVAKLIGCNINELTLTLSKRNMRVRNDTIVQKLTLPQAIDARDALAKS

AtMyo8A NHVEVVADEAVTNVAMLMGCNSKKLMVVLSTCKLQAGRDCIAKRLTLRQATDMRDSLAKI

AtMyo8C NHVEVVADEAVANAAMLMGCNTEELMVVLSTRKLQAGTDCIAKKLTLRQATDMRDGIAKF

. . .*.: .: *: * * .* . .::. : : : **: ** * ** :**

PpMyo8B IYSYLFDWLVERVNKSLEAGKLRTGRSISILDIYGFETFKRNSFEQLCINYANERLQQHF

PpMyo8C IYSYLFDWLVQRVNKSLEVGKTLTGRSISILDIYGFESFQRNSFEQLCINYANERLQQHF

PpMyo8E IYANLFDWLVERINKSLEVGKKRTGRSISILDIYGFESFQKNSFEQLCINYANERLQQHF

PpMyo8A IYASLFDWLVGRINKSLEVGKKPTGRSISILDIYGFESFKKNSFEQLCINYANERLQQHF

PpMyo8D IYASLFDWLVERINKSLEVGKKRTGRSISILDIYGFESFKKNSFEQLCINYANERLQQHF

NbMyo8A IYANLFDWIVDKINKSLAMSQEKTARTINIVDIYGFESFEKNSFEQLCINYANERLQQHF

NbMyo8G IYANLFDWIVDKINKSLAMSKKKTARTIFIVDIYGFESFEKNSFEQLCINYANERLQQHF

NbMyo8C IYANLFDWIVDQMNRKLAMGKEQKGRSINILDIYGFESFKRNSFEQFCINYANERLRQHV

NbMyo8E IYANLFDWIVDQMNRKLAMDKEQKGRSINILDIYGFESFKRNSFEQFCINYANERLQQHV

NbMyo8B IYSCLFDWLIEQINKSLAAGKRRTGRSISILDIYGFESFERNSFEQFCINYANERLQQHF

NbMyo8D IYSCLFDWLVEQINKSFAAGKRRTGRSISILDIYGFESFERNSFEQFCINYANERLQQHF

AtMyo8B IYACLFDWLVEQINKSLAVGKRRTGRSISILDIYGFESFNKNSFEQFCINYANERLQQHF

AtMyo8D IYSCLFDWLVEQINKSLAVGKRRTGRSISILDIYGFESFDKNSFEQFCINYANERLQQHF

AtMyo8A IYASLFNWLVEQINISLEVGNSRTGRSISILDIYGFESFKDNSFEQFCINYANERLQQHF

AtMyo8C IYANLFDWLVEQINIALEVGKSRTGRSISILDIYGFESFKNNSFEQFCINYANERLQQHF

**: **:*:: ::* : : ..*:* *:******:*. *****:*********:**.

PpMyo8B NRHLFKLEQEEYTSEDIDWTRIEFQDNQQCLDLIEKRPVGLISLLDEECMFPRATDFTLA

PpMyo8C NRHLFKLEQEEYTSEDIDWTRIEFEDNQECLDLIEKRPVGLLSLLDEECMFPRATDVTLA

PpMyo8E NRHLFKLEQEEYTSENIDWTRVDFEDNQECLDLIEKRPLGLISLLDEECMFPRSSDLTLA

PpMyo8A NRHLFKLEQEEYTSENIDWTRVDFEDNQECLDLIEKRPLGLISLLDEECMFPRASDATLA

PpMyo8D NRHLFKLEQEEYTSENIDWTRVDFEDNQECLDLIEKRPLGLISLLDEECMFPRASDLTLA

NbMyo8A NRHLFKLEQEEYELDGIDWTKVDFQDNQECLDLFEKKSIGLISLLDEESNFHKATDLTFT

NbMyo8G NRHLFKLEQEEYELDGIAWTKVDFQDNQECLDHFEKKSIGLISLLDEESNFHKATDLTFT

NbMyo8C NRHLLKLEQEEYELDGIDWTKVDFEDNQECLDLFERKPIGLISLLNEESNSLKATDLTFA

NbMyo8E NRHLLKLEQQEYELDGIDWTKVDFEDNQECLDLFEKKPIGLISLLNEESNSLKATDLTFT

NbMyo8B NRHLFKLEQEEYIQDGIDWTKVYFDDNQDCLNLFEKKPLGLLSLLDEESTFPNGTDMSFA

NbMyo8D NRHLFKLEQEEYIQDGIDWTKVDFDDNQDCLNLFEKKPLGLLSLLDEESTFPNGTDMSFA

AtMyo8B NRHLFKLEQEEYIQDGIDWTRVDFEDNQECLSLFEKKPLGLLSLLDEESTFPNGTDLTLA

AtMyo8D NRHLFKLEQEEYIQDGIDWTRVDFEDNQNCLSLFEKKPLGLLSLLDEESTFPNGTDLTLA

AtMyo8A NRHLFKLEQEEYEGDGIDWTKVEFIDNQECLNLIEKKPIGLVSLLNEESNFPKATDTTFA

AtMyo8C NRHLFKLEQEEYEEDGIDWTKVEFVDNQECLDLIEKKPIGLLSLLDEESNFPKATDLTFA

****:****:** : * **:: * ***:**. :*:: :**:***:**. ..:* :::

PpMyo8B NKLKDHLKKNASFRGERDKKFRVYHYAGEVLYEADGFLEKNRDLLHADLVELLESCDCAL

PpMyo8C NKLKDHLKRNASFKGERDKKFRIYHYAGEVLYETDGFLEKNRDLLHADLVEVLRSCDCTM

PpMyo8E NKWKEHLKGNVCFKCERDKAFRVCHYAGEVVYETNGFLEKNRDLLHADLLQLLASCDCAL

PpMyo8A NKLKEHLKGNDCFKGERDKAFRICHYAGEVVYETSAFLEKNRDLLHADLLQLLASCDCAL

PpMyo8D NKLKDHLKGNDCFKVEREKAFRVCHYAGEVVYETNGFLEKNRDLLHSDLLQLLTSCDCEL

NbMyo8A NKLKQHLKANPCYKGDRE-EFGIRHYAGEVIYGTSGFLEKNRDTVHSDIIQLLSSSSEHL

NbMyo8G NKLKQQLEANPCYKGDRE-EFGIRHYAGEVIYDTSGFLEKNRDTVHSDIIQLLSSSSEHL

NbMyo8C SKLQQHIKSDPCFKGERG-EFHIRHYAGEVTYDATGFLEKNRDALHSDIIQLLSSSSGQL

NbMyo8E SKLQQHIKSDPCFKGERE-EFHIRHYAGEVTYDATGFLEKNRHALHSDIIQLLSSSGGQL

NbMyo8B DKLKQHLNSNLCFRGERGKAFTVCHYAGEVTYDTTGFLEKNRDLLHSNSIQLLSSCKYHL

NbMyo8D DKLKQHLNSNLCFRGERGKAFTVCHYAGEVTYDTTGFLEKNRDLLHSNSIQLLSSCKYHL

AtMyo8B NKLKQHLNDNSCFRGDRGKAFTVAHYAGEVTYETTGFLEKNRDLLHSDSIQLLSSCSCHL

AtMyo8D NKLKQHLQSNSCFRGDKGKLFTVVHYAGEVTYETTGFLEKNRDLLHSDSIQLLSSCSCLL

AtMyo8A NKLKQHLNANSCFKGERGRGFRIKHYAGEVLYNTNGFLEKNRDPLHVDLIQLLSLCKCQL

AtMyo8C NKLKQHLKTNSCFKGERGRAFRVNHYAGEVLYDTNGFLEKNRDPLPADLINLLSSCDCQL

.* ::::: : .:: :: * : ****** * : .******. : : :::* . :

PpMyo8B IFDFLASAGQGSGKS----------NGSEYQKQSVASKFKGQLNKLLQRLEATEPHFIRC

PpMyo8C TRQFLA--GQGSQRS----------NGSEYQKQSVAAKFKGQLNKLMQRLEATEPHFIRC

PpMyo8E SQLFAASIGDGVQKL-ISPTRRSFNGSTESQKQSVATKFKGQLNKLMQRLESTEPHFIRC

PpMyo8A PKLFGASIEDGAQKL-LSPNRRA--NGMESQKQSVAAKFKGQLNKLMQRLESTEPHFIRC

PpMyo8D PQLFGASIGDGAQKL-LSPNRRA--NGTESQKQSVAAKFKGQLYKLMQRLESTEPHFIRC

NbMyo8A PKSFA-SFANQ---------------SADFQKQTVATKFKDLLFKLMQQLESTAPHFVCC

NbMyo8G PKSFASSFANQ---------------SIDFQKHTVATKFKTLLFKLMQQLESTAPHFVCC

NbMyo8C PQLFASVSANEDT-EVSSPSTYA--RVPDFQKQSVATKFKDHLFKLMQQLENTTPHFICC

NbMyo8E PQLFASVSANEDT-EFSSPSIYT--GVKDFQKQSVATKFKDHLFKLMQQLENTTLHFICC

NbMyo8B PQTFASYMLAQSEKPVIGPLYKS--GGADSQKLSVSTKFKGQLFQLMQRLETTTPHFIRC

NbMyo8D PQTFASYMLAQSEKPVVGPLYKS--GGADSQKLSVSTKFKGQLFQLKQRLETTTPHFIRC

AtMyo8B PQAFASSMLIYSEKPLVGPLHKA--GGADSQRLSVATKFKGQLFQLMQRLGNTTPHFIRC

AtMyo8D PQAFASSMLIQSEKPVVGPLYKA--GGADSQRLSVATKFKSQLFQLMQRLGNTTPHFIRC

AtMyo8A LNLFSTKMHHDFLKPAT---------FSDSMNQSVIAKFKGQLFKLMNKLEDTTPHFIRC

AtMyo8C LKLFSTKMRGKSQKPLM---------LSDSTNQTVGTKFKGQLFKLMNKLENTSPHFIRC

* : . :* :*** * :* ::* * **: *

PpMyo8B IKPNTQQLPNVIDQKLVLQQLRCCGVLEVVRISRSGYPTRYTHNEFASRYAFLLPRDVSE

PpMyo8C IKPNTQQLPNVIDQKLVLQQLRCCGVLEVVRISRSGYPTRHTHNDFANRYAFLLPRDVSE

PpMyo8E IKPNTSQLPDIFEQGLVLQQLRCCGVLEVVRISRSGYPNRHSHDEFASRYGFLLPRSLSN

PpMyo8A IKPNTSQLPNIFEQDLVLHQLRCCGVLEVVRISRSGYPTRHSHHEFAKRYGFLLPRNLSN

PpMyo8D IKPNASQFPNIFDQKLVIQQLRCCGVLEVVRISRSGYPTRHSHHEFATRYGFLLPRNLSN

NbMyo8A IKPNNKQVPGLYNNDLVFEQLRCSGLLDIVRISRSGYPTRMTHLEFSKRYGVLRPQVHES

NbMyo8G XKPNNKQAPGMYNNDLVFEQLRCSGLLDIVRTSRSGYPTRMTHQEFSKRYGVLRPQVHES

NbMyo8C IKPNNKQVPGMCDKDLIIQQLRSCGVLEVVRISRSGYPTRLTHQEFTSRYGFLLVKDNAC

NbMyo8E IKPNNKQVPGICDKDLVIQQLRSCGVLEVVRLSRSGYPTRLTHQEFTSRYGFLLVKDNAR

NbMyo8B IKPNNFQSPGKYEQGLVLQQLRCCGVLEVVRISRAGFPTRMSHQKFARRYGFLLLDHVAS

NbMyo8D IKPNNFQSPGKYEQGLVLQQLRCCGVLEVVRISRSGFPTRMSHQKFARRYGFLLLDHVAS

AtMyo8B IKPNNVQSAGLYEQGLVLQQLRCCGVLEVVRISRSGFPTRMFHHKFARRYGFLLLENIAA

AtMyo8D IKPNNIQSPGVYEQGLVLQQLRCCGVLEVVRISRSGFPTRMSHQKFSRRYGFLLVENIAD

AtMyo8A IKPNSNQLPGLYEENHVLQQLRCCGVLEIVRISRSGYPTRLTHQELAVRYGCLLLDTRIS

AtMyo8C IKPNSKQLPRVYEEDLVLQQLRCCGVLEVVRISRSGYPTRLTHQEFAGRYGFLLSDKKVA

*** * :: ::.***..*:*::** **:*:*.* * .:: **. *

PpMyo8B QEDVLSVCVAILEHFRKFITSEMYQVGITKLFFRAGQIGMLEDVRVRTLRSIDRAQAVYK

PpMyo8C QEDVLSVCVAILEHFKKYFTSEMYQVGISKLFFRAGQIGMLEDVRVRTLHSIDRAQAVYK

PpMyo8E QEDVLDICVSILHQFG--IPPDMYQVGISKLFFRAGQIGHLEDVRLRTLQGVTRVQAVYK

PpMyo8A QEDMLSICVSILHQFG--IAPDMYQVGITKLFFRAGQIGHLEDVRLRTLQGITRVQALYK

PpMyo8D QEDVLSICVSILHQFG--IAPDMYQVGITKLFFRVGQIGHLEDVRLRTLQSVIRVQALFR

NbMyo8A -KDPLSMSVAILRQFD--ILPEMYQVGYTKLYFRAGQIAALEDVRKQVLQGTLEVPKCYS

NbMyo8G -KDPLSMSVAILRQFD--ILPEMYQVGYTKLYFRAGQIAALEDVRKQVLQXTLEVPKCYS

NbMyo8C -QDPLSMSVAIQQQFD--ILPELYLVGYTKLYFRAGQIAALEDVRNQVLQGTLEVQKCFR

NbMyo8E -QDPLSMSVAILQQFD--ILPELYQVGYTKLYFRVGQIAALEDVRNQVLQGTLEVQKCFR

NbMyo8B -QDPLSVSVAILHQFN--ILPDLYQVGFTKLFFRTGQVGVLEDTRNRTLHGILRVQSFFR

NbMyo8D -QDPLSVSVAILHQFN--ILPDLYQVGFTKLFFRTGQVGVLEDTRNRTLHGILRVQSCFR

AtMyo8B -KDPLSVSVAILHQFN--ILPEMYQVGYTKLFFRTGQIGVLEDTRNRTLHGILRLQSYFR

AtMyo8D -RDPLSVSVAILHQFN--ILPEMYQVGYTKLFFRTGQIGVLEDTRNRTLHGILRVQSSFR

AtMyo8A -QDPLSTSKAILKQCN--LPPEMYQVGYTKIYLRTGVISVLEERKKYVLRGILGLQKQFR

AtMyo8C -QDPLSVSIAVLKQYD--VHPEMYQVGYTKLYLRTGQIGIFEDRRKKVLQGIVGLQKHFR

.* *. . :: .: . ::* ** :*:::*.* :. :*: : .*: :

PpMyo8B GYKVRREYKKKRKAVVFLQSLVRAAIARRHFEKRKERHRAVVFIQKNVRGWIARCAYQAK

PpMyo8C GYKVRRAYKKTRKTIIFLQCLVRSAIARRRFEKIKQTHRAARIIQKQVRRWSARRAYQAK

PpMyo8E GYKARCIYKQRRMTTIILQCMVRGAIARKRFGRLLERHRAAVIVQKYARQQSACRKYQSI

PpMyo8A GYKVRCNYKHRRATTIFLQSLVRGAIARRRFELLRERHRAAVTIQKYARRQVACRRYRSV

PpMyo8D GYKDRCNYKHLRMTTIFVQSMVRGAIARRRFELLQERHRAAVMIQKFARRQVVSRRYQST

NbMyo8A GHCARRHFHELEGGVIILQSFIRGEIARRQYNASLESKRKAA-----------NKENDKQ

NbMyo8G GHCARRHFHELEGGVIILQSFVRGEIARRQYNASLELKRKAA-----------NKENDKQ

NbMyo8C GYRARRYLHELKGGVITLQSFIRGEIARNRYNTSVGSKAKVA-----------HK-SDEQ

NbMyo8E GHRARRYFHELKGGVITFQSFICGEIARNRYNTSVGSKTKVA-----------HK-SDEQ

NbMyo8B GHQARRHLKQLGRGIATLQSFVRGEKARKEYAILLQRHRAALCIQKQIKCRSKRNTYRNI

NbMyo8D GHQARRHLKQLGRGIATLQSFIRGEKARKEYAILLQRHRAALCIQKQIKCRTKRKTYRNI

AtMyo8B GHQARCRLKELKTGITILQSFVRGEKMRKEYTELLQRHRASAAIQSHVKRRIASQQYKAT

AtMyo8D GYQARCLLKELKRGISILQSFVRGEKIRKEFAELRRRHKAAATIQSQVKSKIARIQYKGI

AtMyo8A GYQTREYFHNMRNAAVILQSYIRGENARRNYIVVGE--SAIV-----------STAITKE

AtMyo8C GHLSRAYFQNMRKVTLVLQSYIRGENARRLFDTEAKFHADSV-----------SEASTDE

*: * :. .*. : . *. :

PpMyo8B KEKVILIQSVVRMSLAKGQLNDLQKEAEEKRAVERKLAEE-----------KRASELQLA

PpMyo8C KKNVIMVQSVARMWLAKREFYALQREGEEKRVAEARLAAEKKAAEEKLAEEKRVAEAKLV

PpMyo8E KEKIVKVQAVIRMWLARKQFLAQRREAEERLATEAKLRVEAQAREEA-----RIKEET--

PpMyo8A KENIVILQSVVRMWLSRKQSLARKKE-----ANEAKRAMESKLSEEA-----RVAETE--

PpMyo8D KEKIVRLQSVVRMWLARKQLFSQRREAEKKIASEKKRAMEAKFSEER-----RIAEET--

NbMyo8A LVAVVQIQSAIRCWLAQRHLNQLQSLKKLNQDREKQGRKT----------VE--------

NbMyo8G LVDVVQIQSAIRCWLTQKHRNQLQSLKKLNEDREKQGRKT----------LE--------

NbMyo8C LVAVVQIQSAIRGWLARKDLNKLQSAKTLNVGIPKTGRKM----------K---------

NbMyo8E LVAVVQIQSAIRGWLARKDLNKLQSAKTLNVDIPKTGRKM----------V---------

NbMyo8B HDASIVIQSVIRGWLVRRCSGDIGLLQF--------------------------------

NbMyo8D HDASVVIQSVIRGWLVRRCSGDIGLLQF--------------------------------

AtMyo8B VDASAVIQSAIRGELVRRCAGDIGWLSS--------------------------------

AtMyo8D ADASVVIQSAIRGWLVRRCSGDIGWLKS--------------------------------

AtMyo8A LDAAIHLQYMVRKWLARKLLNSTQQKNKPRNEKKKTRRKS----------TKRV------

AtMyo8C LSAVIHLQSAVRGWLARKHFNSMQRQKELRNVATKSKRKA----------GRRI------

:* * * :

PpMyo8B AEIQEKEAAEEKVRIEAVLQEEVRMRRQAEEGTGSADEEQESIKEICETITTKPPESEEQ

PpMyo8C EEAEEKIAADAKVAFETVLSEEARLTRQA-DGTVSADEEQESIKEIYEMATTKRFESEEQ

PpMyo8E -----------------KLKKERMIHEQHTFADDERDEEPELIKV---------VAAEEL

PpMyo8A -----------------ANVK-----------EDAVDDGRECIKEVASS--TRAESAKEL

PpMyo8D -----------------ESKQDFTTNGKDALPNVEGDGDLECVKEVATP--EPAVFAQVM

NbMyo8A --------------------------------------------------------VKP-

NbMyo8G --------------------------------------------------------VKQ-

NbMyo8C ---------------------------------------------------------VK-

NbMyo8E ---------------------------------------------------------AK-

NbMyo8B ------------------------------------------------------GGRKGN

NbMyo8D ------------------------------------------------------GGRKGN

AtMyo8B ------------------------------------------------------GGTKRN

AtMyo8D ------------------------------------------------------GGAKTN

AtMyo8A ------------------------------------------------------SEDKEL

AtMyo8C ------------------------------------------------------SEDKDI

PpMyo8B NESTIRVRPSHILELQQRAVIAERTLLEKEEDNALLRQRIQHYENQWVEYEAKMSSMEEM

PpMyo8C NEATIRVRPSYVLELQQRAVIAEKALREKEEEIVLQRQKIQHYEKQWAEYEAKISSMEEK

PpMyo8E QEVTIKVRPSYLLELQRRAVMAEKALREKEEENASMRQKILHYEARWMEYEAKMTSMEEM

PpMyo8A KEATIKVAPSYLLELQRRAVMAEKALREKEEDNAMLRQRLLHYEARWMEYEAKMSSMEDM

PpMyo8D KEATIKVAPSYLLELQRRAVMAEKALREKEEDNAVLRQRLLHYEARWMEYEAKMSSMEEM

NbMyo8A -DLPAEILPSVVEDLERRVMVAEASLGEKDKENAALKEQVNQLEARWSDYEVRMRSMEEM

NbMyo8G -ELPAEILPSVVEDLERHVMVAEASLGEKDKENAALKEQVNQLEARWSDYEVRMRSMEEM

NbMyo8C -ELPREILPSVVEDLERRLLKAEATLGEKEMENVALKEQLNLFKARCLEYEVKMRSMEDM

NbMyo8E -ELPREILPSVVKXLEKRLLKAEATLGEKEMENVALKEQLNLFEARCLEYEVKMKSMEEM

NbMyo8B ESEEVLVKSSFLAELQRRVLRAEAALREKEEENDILHQRLQQYENRWSEYELKMKSMEEI

NbMyo8D ESEEVLVKSSFLAELQRRVLRAEAALREKEEENDILHQRLQQYENRWSEYELKMKSMEEI

AtMyo8B ESDEVLVKASYLSDLQRRVLRTEAALREKEEENDILRQRVQQYDNRWSEYETKMKSMEEI

AtMyo8D ELGEVLVKASVLSELQRRVLKAEAALREKEEENDILQQRLQQYENRWSEYETKMKSMEEI

AtMyo8A LSEQFEVQPCVLADLQSRVLKVEAAIMQKEDENTALQEELQRFEERWLENETRMKSMEDT

AtMyo8C PLEQPQVQPTSMSDLQKRILKSEAALSQKEEENTALREQLRQFEERWSEYDIKMKSMEET

: : *: : : * :: :*: : ::.: . : : : :: ***:

PpMyo8B WQKQMSTLQLSLAAAKKSIAT-------------------EESATLQTSSKDG-SEDQKT

PpMyo8C WQKQMSTLHLSLAAAKKSIATEESATLHLNLPAAKKSIATEESATLQTSPEDD-SGDLKT

PpMyo8E WQKQMSSLQLSLSAAKRSLATDDYSM-------------------LQTPTKDHDSINDRF

PpMyo8A WQKQMSSLQLSLAAAKKSLATDEFLP--------------------QTPGKH---DNGRI

PpMyo8D WQKQMSSLQLSIAAAKQSLATDEHPL--------------------QTPVKD---DNGCI

NbMyo8A WQKQMVSLQASLAAAKKSLGVDNPAGHPGKR-------------EGSQSPCGYDSEDT-T

NbMyo8G WQKQMVSLQASLAAAKKSLGVDNPAGHPGKH-------------EGSQSPCGYDSEDT-T

NbMyo8C WQKQMTSLQASLVAAKNSVGAGDTTGRPGKP-------------EGSPSPRYYDSDDA-T

NbMyo8E WQKQMASLQASLAAAKNSLGAGDTTGRPGKP-------------EGSPSPRYYDSDDA-T

NbMyo8B WQKQMRSLQSSLSIAKRSLTLDDS-RNSDAS----------------VNPTDEK-ESS-W

NbMyo8D WQKQMRSLQSSLSIAKRSLTLDDSRRNSDAS----------------VNPTDEK-DSS-W

AtMyo8B WQKQMKSLQSSLSIAKKSLEVEDSARNSDAS----------------VNASDAT---D-L

AtMyo8D WQKQMRSLQSSLSIAKKSLAVEDSARNSDAS----------------VNASDAT---D-W

AtMyo8A WQKHMSSMQMSLAAACKVLAPDKT------------------------ASHGTDSEDT-M

AtMyo8C WQKQMSSLQMSLAAARKSLAAESITGQAGGR------------QDTSISPFGYDSEDT-M

***:* ::: *: * . :

PpMyo8B VAGKHNRNTR-PLLPTEEEKFHKVIQDLDDEAAKVPENVENNSNKFLHAGSELGSSQGEV

PpMyo8C TGGKLSRGTR-PLLPTEEEKFHKDTQELDEESAKVTMDSEQNSNKFLHAGSELGSSQGEV

PpMyo8E SAGKHQRTKRQLLPPPDDEEFD-----WDDATTNGTRSPDQFYNRYLLPGRECSTPRGDV

PpMyo8A SAGKHRHSTKRQLLPSDDEEFD-----WDDVATNGMKSPDDFTNKYLVTGSGNGASRGDV

PpMyo8D SIEKQQRITKRQLLPPGDEQLD-----WDDAATNGTRSPDQFTNKYLVTGSEYSTPRGDV

NbMyo8A TMGTH---------------------------TPGGSTPI---E-FASNGVD-LGGIRGN

NbMyo8G TMGTH---------------------------TPGGSTPI---E-FASNGVD-FAGIRGN

NbMyo8C CMD-----------------------------TLAGCTPV-----KFTDSLG-VGANREV

NbMyo8E SMD-----------------------------TPAGCNPS---X-KFTNSLG-VGANREV

NbMyo8B ETG-----------------------------SNHRA---------R-----ESNGARPM

NbMyo8D ETG-----------------------------SNHRA---------R-----ESNGARPM

AtMyo8B DSG-----------------------------GSHYQ---------MGH-----GRSRSV

AtMyo8D DSS-----------------------------SNQFR---------SQTSNGVGSRLQPM

AtMyo8A SFG-----------------------------TPT----------------------KEL

AtMyo8C STG-----------------------------TPGVRTPT---N-KFTNGNTPELRIREL

PpMyo8B AAGHSYVTQLDREFDHRKQVFTDDIDFLVEVKSGQTTA-----HLSPEDELRKLKTRFDA

PpMyo8C AAGHSYVVQLDREFDHRKQVFTDDIDFLVEVKSGQTQA-----HLNPEDELRKLKTRFDG

PpMyo8E DAARSVVNHLVREFDHRTQVFNDDADFLIEVKSGLTEA-----PLDPEEELRKLRMRFDT

PpMyo8A EAARSVVSHLTREYDHRTQVFNDDVDFLIEVKSGLTEA-----NLNPEEELRKLKVRFDT

PpMyo8D DAARSVVNHLMREYDHRTQVFNDDVDFLVEVKSGLTEA-----HLNPEDELRKLKVRFDT

NbMyo8A NGSLCAVNYLNREFELRRQNFDDEAMAIAQLKSEQLHS------TNPAEDFRRLRHRFEE

NbMyo8G NGSLCAVNYLNREFELRRQNFDDEAMAIAQLKSEQLHS------TNPAEDFRRLRHRFEE

NbMyo8C NGGLAIVSHLTLEFEQRKQNFDDEALAIVHLKPEQLHP------TNPADEYRRLKCRFEE

NbMyo8E NGGLAIVSHLTLEFEQRMQNFDDEALAIVHLKPGQFHS------TNPADEYRRLKCRFDE

NbMyo8B NAGLSVISRLAEEFEQRSQVFGDDAKFLVEVKSGQVEA-----NLNPDHELRRLKQMFEA

NbMyo8D SAGLSVISRLAEEFEQRSQVFGDDAKFLVEVKSGQVEA-----NLNPDRELRRLKQMFEA

AtMyo8B GVGLSVISRLAEEFGQRAQVFGDDRKFLMEVKSGQVEA-----NLNPDRELRRLKQMFET

AtMyo8D SAGLSVIGRLAEEFEQRAQVFGDDAKFLVEVKSGQVEA-----NLDPDRELRRLKQMFET

AtMyo8A KGSLSDVNNLSTEFDQRSVIIHEDPKSLVEVKSDSISN-----RKQHAEELRRLKSRFEK

AtMyo8C NGSLNAVNHLAREFDQRRLNFDEDARAIVEVKLGPQATPNGQQQQHPEDEFRRLKLRFET

. : * *: * : :: : .:* : *:*: *:

PpMyo8B WKKDFKVRLRETKAVLSKLGHTDSSDKW---------------IRGKKWHWVKLGKQITP

PpMyo8C WKRDFKVRLRETKVVLNKLSHTDSTDRW---------------IRGKKWHWGKLGKQVAP

PpMyo8E WKKDFKTRLRETKLVLQRLCNVDSAE----------------KEKTRKKWWSK---RTTP

PpMyo8A WRRDFKARLRETRLVLNKLCSLDSAEKDGDRMLCALDSLEKEGDRTRKKWWGK---KTTS

PpMyo8D WKKDFKVRLRETKLVLNKLCAMDSAE--------------KEKDRTRRNWWGK---RTTP

NbMyo8A WKKDYKARLKETKAKVHKFGYSE----A---------------EKTRRNWWGKKSKR---

NbMyo8G WKKDYKARLKETKAKVHKFGYSE----A---------------EETRRNWWGKKSKR---

NbMyo8C WKKDYKVRLKETKAKVHKLGCSK----A---------------GKNRRKWWGKKSK----

NbMyo8E WKKDYKVRLKETKAKVHKLGCSK----A---------------GKNRRKWWGKKIK----

NbMyo8B WKKDYGSRLRETKVILNKLGSDE--GAS---------------DKMKKKWWGRRNSTRFN

NbMyo8D WKKDYGSRLRETKVILNKLGSDE--GAS---------------DKMKKKWWGRRNSTRFN

AtMyo8B WKKDYGGRLRETKLILSKLGSEETGGSA---------------EKVKMNWWGRLRSTRY-

AtMyo8D WKKDYGGRLRETKLILSKLGSEESSGSM---------------EKVKRKWWGRRNSTRY-

AtMyo8A WKKDYKTRLRETKARV-RLNGDE---------------------GRHRNWWCKKSY----

AtMyo8C WKKDYKARLRDTKARLHRVDGDK---------------------GRHRKWWGKRG-----

*::*: **::*: : :. . : * :

PpMyo8B P-----

PpMyo8C P-----

PpMyo8E ------

PpMyo8A SRALQG

PpMyo8D ------

NbMyo8A ------

NbMyo8G ------

NbMyo8C ------

NbMyo8E ------

NbMyo8B ------

NbMyo8D ------

AtMyo8B ------

AtMyo8D ------

AtMyo8A ------

AtMyo8C ------
