# Supplementary material for: Myosin VIII associates with microtubule ends and together with actin plays a role in guiding plant cell division
Source: eLife. 2014 Sep 23;3:e03498. doi: 10.7554/eLife.03498 (PMC4171706; doi:10.7554/eLife.03498)
Supplement: Figure 4—source data 2. — DOI: http://dx.doi.org/10.7554/eLife.03498.013 [file elife03498s002.docx]

Myosin VIII Amino Acid Sequence Comparison

|  | PpMyo8B | PpMyo8C | PpMyo8E | PpMyo8A | PpMyo8D | NbMyo8A | NbMyo8G | NbMyo8C | NbMyo8E | NbMyo8B | NbMyo8D | AtMyo8B | AtMyo8D | AtMyo8A | AtMyo8C |
| --- | --- | --- | --- | --- | --- | --- | --- | --- | --- | --- | --- | --- | --- | --- | --- |
| PpMyo8B | 100 | 78.68 | 61.24 | 62.2 | 62.29 | 46.66 | 46.19 | 49.12 | 48.45 | 53.07 | 52.89 | 52.26 | 52.81 | 49.55 | 51.13 |
| PpMyo8C | 78.68 | 100 | 61.49 | 61.98 | 62.43 | 46.41 | 45.86 | 48.02 | 47.28 | 52.57 | 52.17 | 51.95 | 52.06 | 49.11 | 51.17 |
| PpMyo8E | 61.24 | 61.49 | 100 | 73.67 | 73.56 | 48.06 | 47.42 | 48.19 | 47.97 | 55 | 54.86 | 54.79 | 55.17 | 50.09 | 50.93 |
| PpMyo8A | 62.2 | 61.98 | 73.67 | 100 | 80.71 | 49.87 | 49.48 | 50.09 | 49.51 | 54.51 | 54.64 | 55.18 | 55.38 | 50.45 | 51.74 |
| PpMyo8D | 62.29 | 62.43 | 73.56 | 80.71 | 100 | 49.05 | 48.41 | 50.22 | 50.09 | 54.11 | 54.33 | 54.78 | 54.46 | 50.63 | 52.24 |
| NbMyo8A | 46.66 | 46.41 | 48.06 | 49.87 | 49.05 | 100 | 95.74 | 69.9 | 69.76 | 52.42 | 52.56 | 53.1 | 53.22 | 52.2 | 56.31 |
| NbMyo8G | 46.19 | 45.86 | 47.42 | 49.48 | 48.41 | 95.74 | 100 | 68.79 | 68.47 | 51.83 | 51.87 | 51.77 | 52.27 | 50.99 | 55.58 |
| NbMyo8C | 49.12 | 48.02 | 48.19 | 50.09 | 50.22 | 69.9 | 68.79 | 100 | 93.43 | 53.9 | 54.4 | 52.91 | 54.2 | 54.22 | 57.86 |
| NbMyo8E | 48.45 | 47.28 | 47.97 | 49.51 | 50.09 | 69.76 | 68.47 | 93.43 | 100 | 53.34 | 53.85 | 52.17 | 53.28 | 53.94 | 57.89 |
| NbMyo8B | 53.07 | 52.57 | 55 | 54.51 | 54.11 | 52.42 | 51.83 | 53.9 | 53.34 | 100 | 97.91 | 75.66 | 76.86 | 54.53 | 55.1 |
| NbMyo8D | 52.89 | 52.17 | 54.86 | 54.64 | 54.33 | 52.56 | 51.87 | 54.4 | 53.85 | 97.91 | 100 | 75.42 | 76.97 | 54.44 | 54.78 |
| AtMyo8B | 52.26 | 51.95 | 54.79 | 55.18 | 54.78 | 53.1 | 51.77 | 52.91 | 52.17 | 75.66 | 75.42 | 100 | 85.29 | 55.02 | 55.5 |
| AtMyo8D | 52.81 | 52.06 | 55.17 | 55.38 | 54.46 | 53.22 | 52.27 | 54.2 | 53.28 | 76.86 | 76.97 | 85.29 | 100 | 55.19 | 56.36 |
| AtMyo8A | 49.55 | 49.11 | 50.09 | 50.45 | 50.63 | 52.2 | 50.99 | 54.22 | 53.94 | 54.53 | 54.44 | 55.02 | 55.19 | 100 | 70.14 |
| AtMyo8C | 51.13 | 51.17 | 50.93 | 51.74 | 52.24 | 56.31 | 55.58 | 57.86 | 57.89 | 55.1 | 54.78 | 55.5 | 56.36 | 70.14 | 100 |

Numbers indicate percent identity.

*Physcomitrella patens*, Pp; *Nicotiana benthamiana*, Nb; *Arabidopsis thaliana*, At
